# The Effect of Medium-Term Sauna-Based Heat Acclimation (MPHA) on Thermophysiological and Plasma Volume Responses to Exercise Performed under Temperate Conditions in Elite Cross-Country Skiers

**DOI:** 10.3390/ijerph18136906

**Published:** 2021-06-27

**Authors:** Ilona Pokora, Ewa Sadowska-Krępa, Łukasz Wolowski, Piotr Wyderka, Anna Michnik, Zofia Drzazga

**Affiliations:** 1 Department of Physiological-Medical Sciences, Institute of Sport Sciences, The Jerzy Kukuczka Academy of Physical Education in Katowice, Mikołowska 72a, 40-065 Katowice, Poland; e.sadowska-krepa@awf.katowice.pl; 2 Doctoral Studies, The Jerzy Kukuczka Academy of Physical Education in Katowice, Mikołowska 72a, 40-065 Katowice, Poland; wolowski.lukasz@gmail.com (Ł.W.); pwyderka90@gmail.com (P.W.); 3 The Silesian Centre for Education and Interdisciplinary Research, Faculty of Science and Technology, University of Silesia in Katowice, 75 Pułku Piechoty 1A, 41-500 Chorzow, Poland; anna.michnik@us.edu.pl (A.M.); zofia.k.drzazga@gmail.com (Z.D.)

**Keywords:** sauna baths, exercise, physiological strain, body temperature, plasma volume, athletes

## Abstract

The influence of a series of ten sauna baths (MPHA) on thermophysiological and selected hematological responses in 14 elite cross-country skiers to a submaximal endurance exercise test performed under thermoneutral environmental conditions was studied. Thermal and physiological variables were measured before and after the exercise test, whereas selected hematological indices were studied before, immediately after, and during recovery after a run, before (T1) and after sauna baths (T2). MPHA did not influence the baseline internal, body, and skin temperatures. There was a decrease in the resting heart rate (HR: *p* = 0.001) and physiological strain (PSI: *p* = 0.052) after MPHA and a significant effect of MPHA on systolic blood pressure (*p* = 0.03), hematological indices, and an exercise effect but no combined effect of treatments and exercise on the tested variables. A positive correlation was reported between PSI and total protein (%ΔTP) in T2 and a negative between plasma volume (%ΔPV) and mean red cellular volume (%ΔMCV) in T1 and T2 in response to exercise and a positive one during recovery. This may suggest that MPHA has a weak influence on body temperatures but causes a moderate decrease in PSI and modifications of plasma volume restoration in response to exercise under temperate conditions in elite athletes.

## 1. Introduction

Heat acclimation (HA) is a method of increasing an athlete’s efficiency for training and competition activity in hot [1,2,3,4] and thermoneutral conditions [2,5,6,7,8]. Heat acclimation is the effect of systematic, artificial exposure of the body to frequent, continuous, or intermittent heat [9], which has an impact on physiological and hematological indices of individuals and can induce numerous physiological adjustments [1,10,11], including: a reduction in resting core body temperature [10,12,13,14], resting heart rate [6,15], an increase in cutaneous heat loss, a greater sweat rate and skin blood flow, as well as lower core temperature thresholds for activating thermoeffectors [4,16]. Moreover, heat acclimation could improve heat tolerance, maximal oxygen uptake [5,17,18,19,20,21,22,23], ergogenic potential for endurance performance [24], energy efficiency of muscle work as well as limit excess fluid loss from the body, feelings of discomfort, and a disturbed relationship between oxygen consumption and the heart rate [4].

Expansion in blood and plasma volumes is a very early change, widely observed and prominent in the HA-phenotype, irrespective of the method employed to adapt to the heat [2,12,25,26,27,28,29,30]. Expansion of blood plasma volume (PV) may contribute to an improvement in the cardiovascular [5,20,31] and thermoregulatory reactions [8,16,28,32], all of which translate into improved physical performance in all (i.e., cool, temperate, and hot) environmental conditions [5,31] and may be translated into a reduction in physiological strain during work [33,34,35].

In athletes, heat acclimation is typically implemented as a training mesocycle immediately prior to competition in order to induce many physiological, cellular, and perceptual adaptations that enhance an individual’s ability to tolerate heat stress [36]. There are many HA protocols [37] that can elicit “optimal” adaptations when the deriving impulses for heat adaptation are associated with inducing thermophysiological strain of a magnitude above an adaptation threshold [4] or as a cumulative adaptation impulse [38]. Passive and active heat stress strategies and their combination are used. The passive HA strategies include resting in a heat chamber [39,40], sauna [15,41,42,43], or hot bath [44,45,46], all of which raise and maintain a moderately high core and skin temperature [32,36]. The protocols from the mentioned studies did not include exercise but made use of different heat stimuli temperatures, durations, and methods. This could potentially explain the conflicting observations of their effectiveness in provoking adaptive changes in the body. The mode of heat acclimation (exercise heat acclimation, passive air, or heat water immersion (HWI)) may affect the type and volume of adaptations gained, due to the medium (air or water) or the nature of the heat stress (active or passive) [47].

Medium-term HA (8–14 days of heat exposure) has been commonly studied in the literature with typical adaptations recently documented in the meta-analysis by Tyler et al. [4]. Sauna bathing has been proposed as one of the most effective passive interventions incorporated into an athletic training program to reduce physiological strain and to improve exercise performance [4]. Positive effects of adaptation to passive isolated hyperthermia are confirmed by the experience of regular (1–2 times a week) prolonged application of dry-air or infrared saunas [32,48,49]. Laboratory-based heat acclimation translates into plasma volume (PV) expansion occurring within the first few days of exposure [20]. This increase in plasma volume could be accompanied by changes in total circulating proteins [26,50].

Expansion of plasma volume (hypervolemia) has been induced in men of various training statuses (healthy, active, well trained, and competitive) following heat chamber, sauna, and HWI protocols with 40–120-min heat exposures [39,41,43,46,51]. Scoon et al. [41] implemented sauna bathing (>50 °C) immediately following training on 12–15 occasions over 3 weeks and demonstrated a 7.1% increase in PV and a 1.9% estimated improvement in 5 km time-trial performance compared with a 3-week period of normal training. Stanley et al. [43] applied sauna bathing following normal training of male cyclists and observed largely expanded PV in well-trained cyclists after just four exposures and concluded that using post-exercise sauna bathing may offer a time-efficient means to stimulate heat acclimation without substantial impact on athletes’ daily training. Zapara et al. [23] applied a protocol of prolonged passive whole-body intense (temperature in the thermo-capsule increased up to 75–80 °C) hyperthermia to test thermal adaptation effects on exercise efficiency in elite athletes and showed that prolonged adaptation to passive hyperthermia (without exercising) leads to increasing the aerobic capacity of men–amateur athletes tested under thermal-neutral conditions.

The heat-induced PV expansion is sometimes difficult to indicate for highly trained endurance athletes because they already possess high PV in accordance with their training status and the mode of exercise training [10]. This is due to the fact that endurance-trained athletes exhibit some features as if they were heat acclimated [4], and highly trained individuals have already developed some thermal adaptations resulting from their training history [52], which might limit the efficiency of HA protocols for inducing further adaptations. Therefore, passive, sauna-induced heat acclimation may be ineffective in athletes in achieving typical adaptive changes, and it may not be manifested during the effort performed under thermoneutral conditions when there are no restrictions to heat dissipation from the body.

Cross-country skiing is a very demanding endurance sport. In the annual training cycle, the period of rest and recovery for athletes is very short. The inclusion of a series of 10 sauna baths in the transitional period of a training program in elite athletes (low training volume + sauna baths) can be an effective way to strengthen and accelerate regeneration and improve the elimination of fatigue and, in the transitional phase of training, can also be used as “a tapering tool” when the training volume is reduced in athletes.

Considering the effects of passive, sauna-induced heat acclimation on physiological responses to exercise in humans, we hypothesized that sauna bathing interspersed across typical training in the transition phase of an annual training program can induce some positive heat adaptations, reduce physiological strain during exercise, and optimize restoration of plasma water during recovery.

Thus, the aims of this study were twofold: (i) to investigate the effects of repeated sauna bathing on heat acclimation adaptations indices, such as internal (Tty) and skin (Tsk) temperatures, the heart rate (HR), and plasma volume in elite athletes, and (ii) to examine if repeated sauna bathing can affect physiological responses to exercise and the plasma water shift during exercise and recovery in elite cross-country skiers in thermoneutral conditions.

## 2. Material and Methods

### 2.1. Ethical Approval

The procedures for this research were approved by the Human Research Ethics Committee (the Research Ethics Committee at the Academy of Physical Education in Katowice, Poland, approval number U2/2016), in accordance with the regulations of the National Health and Medical Research Council (Poland) and in compliance with the Declaration of Helsinki. Prior to the study, all participants gave their written consent to be involved in the study and submitted the Athlete Biological Passport (ABP) for review.

### 2.2. Participants

Sixteen male professional cross-country skiers participated in this study. The inclusion criteria required that all participants had a professional training history of at least ten years. The characteristics and the anthropometric data of the recruited participants are shown in Table 1.

Experiments were conducted from April to June during the transition phase of an annual training program (the average environment temperature was 10–18 °C). In the transition phase of the annual training program, they recorded the duration and intensity of their training prior to the experiment and were instructed to attempt to repeat this training throughout the study. During the sauna period, daily training loads were similar; however, the duration of the training was reduced. During the studies, participants were instructed to consume their normal diet (a mixed diet) and fluid intake as well as regularly provide their first morning urine samples to the laboratory to check for adequate hydration (urine specific gravity < 1.03) [53]. Participants were instructed to enrich as much as possible their diet of fresh raw fruits and vegetables and steamed starchy vegetables for 3 days before the study was conducted. Exercise and heat exposure leads to a marked reduction in plasma volume and an increase in plasma osmolality that is due at least in part to hypohydration resulting from sweat loss. Athletes maintained euhydration through-out HA by drinking ad libitum and replaced exercise related body weight losses up to 150% before the next training session.

### 2.3. Experimental Design

**Figure 1:**
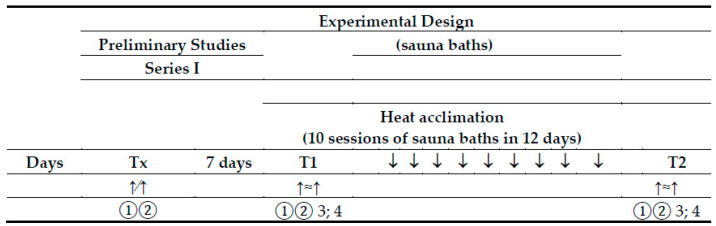
Experimental design. Tx—a preliminary study; T1—experimental exercise test performed before sauna bathing; T2—experimental exercise test performed after a series of ten sauna baths; ↑∕↑—a graded exercise test; ↑≈↑—a submaximal, endurance exercise test; 1—physiological measures and blood collection at rest; 2—physiological measures and blood collection immediately after the exercise test; 3—blood collection at 60 min of recovery; 4—blood collection 24 h after the exercise test.

The experimental procedures were a replication of those previously used in our study [54], with subtle differences noted below and with cross-references provided for additional details (Figure 1).

#### 2.3.1. Preliminary Session

Before the first trial, all subjects completed a familiarization session, a graded exercise test (GTX) to volitional fatigue (Tx), and an anthropometric assessment. During the first visit, one week prior to the start of the experiment, blood samples and anthropometric assessments were taken (Tx), and a graded exercise test was performed. Body mass (BM) and body composition (BMI—body mass index; FM—total body fat; FFM—total fat free mass; TBW—total body water) were assessed (using bioimpedance analysis, Inbody 220, Korea) to describe the anthropometric characteristics of the study participants. During the GXT test, the heart rate (HR), oxygen uptake (VO_2_), and blood lactate concentration were recorded. HR was monitored with a pulse meter Sport-tester (Polar-1500, Finland). Oxygen uptake (VO_2_) was measured using an open-circuit gas analyzer (Cortex Metamax, Germany). Capillary blood samples for determination of blood lactate (LA) were withdrawn from the fingertip before exercise and during the last minute of each exercise work load and every minute until the 6th minute of recovery. Capillary blood lactate levels were determined enzymatically using commercial enzyme kits (Boehringer, Manheim, Germany). Based on blood lactate concentrations, the anaerobic threshold was determined by the D-max method. HR corresponding to anaerobic threshold (AT) exercise intensity was determined (HR-AT). HR-AT was used to appropriate the exercise intensity estimation, which was used accordingly during the experimental tests. This standardization ensured that each participant was required to performed exercise in proportion to his anaerobic threshold, and it resulted in equivalently elevated systemic functions that were then held constant over the 60 min of exercise.

#### 2.3.2. Heat Acclimation—Finnish Sauna Treatments

Experiencing the identical heating protocol. The procedure of Finnish sauna treatments was carried out in a sauna chamber located at Jerzy Kukuczka Academy of Physical Education in Katowice. Athletes completed medium-term heat adaptation (MPHA) consisting of ten Finnish sauna baths with a two-day break between 5 and 6 heat exposures. Missing 1–2 days of exercise-heat exposures during a 10–14 day acclimation period is not likely to impede heat adaptation [14,55]. Participants spent a total of 45 min in the sauna (an average temperature at face height 90 ± 2 °C, and an average relative humidity 12 ± 4%). This total time was divided into three equal parts (each ~15 min) and separated by two 4–6-min showers to cool the body [42,51]. Sauna baths took place in the afternoon after daily training sessions in the transition phase. We decided to standardize the duration of sauna exposure rather than the duration of heat stress (internal temperature > 38.5 °C) to minimize the potential impact on the athletes’ daily life/training schedule. By the end of the procedures, the temperature of an athlete’s body core increased by 1.0–1.5 °C. It is recognized that to launch the thermoregulatory adaptive processes significant for adaptation to metabolic stressors (loads), it is necessary to increase the body core temperature by 1.3–1.5 °C [30]. During and after the heating protocol, the subjects were allowed to consume mineral water with an average mineral content ad libitum.

#### 2.3.3. Standard Submaximal Exercise Test

The main physical effort used in this study was one hour of uninterrupted running exercise (EET) performed in thermoneutral conditions. The participants performed the continuous EET test twice: before (T1) and after a series of 10 sauna baths (T2). The EET test was performed at the predetermined intensity, below HR-AT for 60 min. Two similar exercise tests, at a given intensity, allowed for determining the subjects’ response to similar physical efforts before and after MPHA. During these exercise tests, subjects were running at a steady speed, corresponding to 85–88% of HR-AT. No fluid was consumed during the exercise test. All exercise tests were performed in the Research Center for Sports, Academy of Physical Education in Katowice, under thermoneutral conditions (an ambient temperature of 21–24 °C and a relative humidity of 45–55%) at the same time of day (8.00–14.00) to minimize the circadian rhythm effects, before and after 10 sauna treatments. In addition, exercise testing was performed during the morning under thermoneutral conditions. It is known that the ability to perform prolonged exercise is the same in the morning and afternoon in a neutral environment [56]. Participants were running in shorts and wearing a chest harness, required as protection from falls. The ambient conditions were identical and stable within and among trials. The constant (below AT) work rate was chosen in combination with the temperate condition to provide a compensable thermal state where vasomotor-mediated, dry-heat losses and evaporative cooling could satisfy the heat loss requirement. Subjects acted as their own controls. Twenty-four hours prior to each submaximal test, participants followed a similar diet, abstained from alcohol and caffeine consumption, and completed team training as prescribed.

#### 2.3.4. Training

This study was performed during the transition phase of the annual training program (during the regeneration micro cycle). During the pre-sauna control period, training sessions lasted 73 ± 8 min/day and were performed 5 times per week, with intensity that can be described by the following distribution: 72% from range I (low-aerobic/active recovery), 17% from range II (moderate), 8% from range III (moderate intensity), 3% from range IV (vigorous intensity, submaximal), and 0% from range V (maximal intensity). During the sauna period, training sessions were shorter (52 ± 7 min), but they were of similar frequency (5 times a week) and similar intensity. This protocol was designed to mimic as closely as possible the conditions of a normal training session during the transition phase. During this training phase, training volume is low and averages 60–80 min a day. In T1, more emphasis was placed on the typical training, which accounted for 100% of the total training time. In T2, the typical training session corresponded to ~50%, and the external heat load accounted for ~50% of the total training time/day.

### 2.4. Measures

During EET running tests, oxygen uptake was measured at rest (baseline) and during exercise at 10–20 min and 50–60 min using a metabolic system (Cortex Metamax, Germany), and the data were averaged over this period. Heart rate (HR) was recorded telemetrically at 10-min intervals via Polar-1500, Finland. Internal temperature (tympanic temperature; Tty) along with skin temperatures taken at three different sites (chest (Ts_Ch_), forearm (Ts_F_), thigh (Ts_Th_), and the body mass (BM) (in kg to the nearest 5 g (Inbody 220, Korea) were measured before and after the exercise test.

#### 2.4.1. Internal and Skin Temperatures and Blood Pressure

Internal body temperature was measured from the auditory canal using an insulated, ear-molded plug and thermistor inserted into the external auditory canal close to the tympanic membrane and insulated from the external environment with cotton wool. The thermistors used to measure Tty were accurate to +0.05 °C within the range 30–40 °C (Ellab, E-val-Flex model 1.38, Denmark). This method isolates the auditory canal from the ambient environment and thereby minimizes cutaneous thermal artefacts [28]. Furthermore, these procedures permit the index to closely track esophageal temperature in temperate conditions (25 °C; [57]), with a minimal baseline offset (0.05 °C). When used in this manner, the auditory canal temperature is known to track esophageal temperature [58].

Skin temperatures were recorded using a Tele-Thermometer (Raytek, model 34, Gdańsk, Poland).

Blood pressure, systolic (SDP) and diastolic (DBP), was measured by the researcher using a stethoscope and a sphygmomanometer at rest and immediately after exercise in T1 and T2.

#### 2.4.2. Calculations

Mean body temperature (Tb) was calculated using a formula by Stolwijk and Hardy [59]. Mean skin temperature (Tsk) was calculated as described by Burton [60]. Changes in mean body temperature (ΔTb) and mean skin temperature (ΔTsk) were derived as the difference between values obtained in the final 5 min of exercise and those from the pre-exercise rest periods. To determine physiological strain during exercise in the T1 and T2 exercise test, the physiological strain index (PSI) was calculated. The PSI was calculated from resting Tty and HR compared to the last exercise time point using the equation derived from Moran et al. [34] with the index range from 0 to 10: 1–2 (no/little heat strain), 3–4 (low heat strain), 5–6 (moderate heat strain), 7–8 (high heat strain), and 9–10 (very high heat strain). The PSI reflects combined cardiovascular and thermoregulatory strain on a universal scale; therefore, the fractional cardiovascular and thermoregulatory systems contributions to the physiological strain were calculated according to Pokora and Żebrowska [61].

#### 2.4.3. Blood Sample Collection and Analyses

Blood was collected at four time points during two main exercise tests (T1—before sauna baths and T2—after a series of ten sauna baths): at rest (before the exercise, baseline—“t0”), immediately after the exercise (“t1”), after 1h (“t2”) and 24 h (“t3”) of recovery, and prior to the preliminary study (Tx—during a typical training process in the transition phase of an annual training program). Blood samples were taken from the antecubital vein into the vacutainer tubes with K2-EDTA. Immediately after collection, the samples were divided into two portions. One portion was used for hematological measurements determined using a Sysmex XE2100. These blood samples were analyzed within 48 h of collection using a Sysmex XT-2000 for hemoglobin concentration (Hb), hematocrit (HCT), and mean red cell volume (MCV) (Sysmex, Norderstedt, Germany) at a diagnostic laboratory (Katowice, Poland). The other portion was immediately placed in a centrifuge to separate plasma from whole blood. The separated plasma was stored at −20 °C until further biochemical analysis. Plasma was assayed for protein content TP (using the biuret method (Randox) by means of the Hitachi 917 Modular P analyzer). Plasma osmolality (OsM) was measured by the OS3000 Marcol, Poland, Osmometer and expressed as the number of milliosmoles of solute per kilogram of plasma water. The total plasma protein concentration and plasma osmolality were analyzed within 48 h of collection at the Biochemical Laboratory (Academy of Physical Education in Katowice). The measurement of serum osmolality is relevant to changes in the intracellular and extracellular balance, as a trusted and valuable indicator of solute concentration in the blood. The reference standard was directly measured, and serum/plasma osmolality was categorized as hydrated (275–295 mOsm/kg), impending dehydration (295–300 mOsm/kg), or currently dehydrated (>300 mOsm/kg) [62].

#### 2.4.4. Calculation of Plasma Volume

Percentage changes in PV (%ΔPV) were calculated using the formula proposed by Strauss et al. [63]. The differences in %ΔPV and %ΔTP as well as %ΔOsM, %ΔMCV, and %ΔHCT were calculated: at T2–T1 for the effect of a series of ten sauna baths, and at T1 and T2 for t0–t1 exercise response, t2–t0 1 h recovery, t3–t0 24 h recovery after the exercise test at T1 and T2, respectively.

Total circulating protein (TPP) was obtained from (TP) (1 + ΔPV(%)/100) [64]. The control of fluid and PV depends on the hormonal system and, as was suggested in previous studies, on plasma proteins; therefore, to study the role of plasma proteins, the difference in %ΔPV and %ΔTP was calculated [65]. If there is no loss or gain of intravascular plasma proteins, the expected value of the above-mentioned difference is zero. A significant value >0 can be interpreted as a gain of plasma proteins into the vascular space, whereas a significant value <0 can be interpreted as a loss of plasma proteins out of the vascular space [65,66].

### 2.5. Data Analysis and Statistics

Statistical analysis was performed using the Statistica software package for Windows® (version 13.1, StatSoft, Polska). For all measures, descriptive statistics were calculated. Descriptive statistics in the text are reported as raw means ± SD. When the data did not fulfill the normality of distributions, they were reported as median (the 25th and 75th percentiles).

The Shapiro–Wilk test was used to check the normality of distributions of the studied variables. Student paired t-test was used to compare parameters at the same stage in T1 exercise sessions and after sauna treatments T2. Two-way analysis of variance (ANOVA; intervention × exercise) or one-way analysis of variance (ANOVA; intervention × time) with repeated measures was used to assess differences in thermal, physiological, and hematological measures. The homogeneity of variances in the analyzed groups was verified by Leven’s test. Mauchly’s test for sphericity was included as part of the procedure. When the data did not fulfill the assumptions required for a parametric test, nonparametric Friedman’s ANOVA test was applied). When ANOVA identified a significant difference, Tukey’s post hoc test was used to identify differences between T1 and T2. The level of statistical significance was set at *p* < 0.05; results with *p* < 0.1 were interpreted as tendencies. Friedman’s ANOVA analysis of variance by ranks as well as Wilcoxon test (for intra-group comparisons) and Mann–Whitney test (for inter-group comparisons) were used to determine %ΔPV, %ΔTP, %ΔOSM, %ΔHCT, and %ΔMCV for changes in biomarkers. Effect sizes for main effects and interactions are presented as partial eta squared (ŋ^2^p) (effect size: 0.01 small, 0.06 medium, 0.14 large), while Cohen’s d was used to evaluate differences between two related samples (ES, Cohen’s d: ≥0.2 small, ≥0.5 moderate, ≥0.8 large effect) [67]. The differences were considered significant at *p* < 0.05. The relationships between the variables were expressed as Spearman’s rank correlation coefficient (denoted as r).

## 3. Results

### 3.1. Participants

Well-trained cross-country skiers took part in the experiment during the transition period (TP) of the annual training cycle. During TP, the cross-country skiers were subjected to strict control regarding their diet, exercise, and recovery by a team of experts, i.e., coaches, a physician, and two sport dieticians. The subjects’ main characteristics are presented in Table 1 and Table 2.

### 3.2. The Effect of Heat Acclimation on Physiological and Hematological Variables at Rest

A series of ten sauna baths did not significantly affect either the initial physiological variables (internal and skin temperatures) or body mass. The effect of MPHA intervention on physiological functions at rest was only noted for the heart rate (*p* = 0.001, ES = 1.08), SDP (*p* = 0.04, ES = 0.96). Passive acclimation clearly reduced the heart rate (by ~8 bs/min) but did not significantly influence the tympanic, body, and local skin temperatures (Table 3).

After a series of ten sauna baths, a change in hematological values was noted. We found that after a series of sauna baths hemoglobin concentration (*p* = 0.06, ES = 0.44), TP concentration (*p* = 0.03, ES = 0.58), and HCT (*p* = 0.26, ES = 0.50) were lower at rest, whereas MCV (*p* = 0.03, ES = 0.41) and OsM (*p* = 0.06, ES = 0.56) were higher compared to the control conditions. In addition, a relatively moderate increase in resting PV was noted (+7.42 ± 18.4%) following saunas, while OsM (*p* = 0.06, ES = 0.56) was generally reported at a stable level. At rest plasma osmolality and body mass did not differ between trials (T1 and T2), indicating that subjects began each trial in a similar hydration state.

### 3.3. The Effect of Heat Acclimation on Physiological Responses to Exercise

During both exercise interventions (before and after a series of sauna baths), mean relative exercise intensities throughout EETs were similar across T1 and T2 (T1: 59.5 ± 6.1 vs. T2: 60.05 ± 4.9) % power max (*p* = 0.19, ES = 0.39) (Table 3).

There was no main effect of treatments for internal temperature Tty (*p* = 0.11) and local skin temperatures or mean skin temperature Tsk (*p* = 0.45). The main effect of exercise was proved for Tty (*p* = 0.006, η^2^*p* = 0.58) and $\bar{T}$sk (*p* = 0.001, η^2^*p* = 0.30), but there was no evidence of an interaction effect (Table 3). Tty temperature increased during exercise in all subjects, but the end (last Tty) temperature elicited in an exercise session was similar in T1 and T2 (*p* = 0.11) (Table 3). Average ΔTty during exercise tended to be lower after MPHA vs. T1 (*p* = 0.06) (Table 4).

There was a trend for the main effect of sauna baths for the mean body temperature (*p* = 0.054, η^2^*p* = 0.18). Baseline $\bar{T}$b was lower during T2 (35.5 ± 0.4 °C) than T1 (35.6 ± 0.7 °C) (*p* = 0.53). There was an effect of exercise (*p* = 0.00, η^2^*p* = 0.65), but no evidence of an interaction effect for $\bar{T}$b (*p* = 0.8). Last $\bar{T}$b temperature elicited in an exercise session was slightly higher in T1 than T2 (*p* = 0.17). Average Δ$\bar{T}$b during the exercise tended to be greater in T2 vs. T1 (*p* = 0.24) (Table 4).

There was no main effect of sauna for the mean skin temperature (*p* = 0.45), but there was a main effect of exercise (*p* = 0.001, η^2^*p* = 0.30). There was no evidence of an interaction effect for $\bar{T}$sk (*p* = 0.83). The mean skin temperature was lower in T2 (32.43 ± 0.75 °C) compared with T1 (32.8 ± 1.22 °C); *p* = 0.25 (Table 3).

There was a main effect of treatments for the heart rate (*p* = 0.006, η^2^*p* = 0.44). There was an effect of exercise (*p* = 0.001, η^2^*p* = 0.95) but no interaction effect (*p* = 0.57) for HR. The heart rate measured during the last exercise workload was higher in the control trial than T2 (~5 bpm) (*p* = 0.001, ES = 1.23).

As noted in Table 4, the participants in the T2 and T1 trials experienced body mass loss during the exercise test. This body mass loss during the T1 and T2 trials was similar (*p* = 0.19).

No significant PSI differences were found for the matched experimental model between the T1 and T2 groups, but the PSI tended to be lower in T2 than T1 conditions (*p* = 0.052, ES = 0.87). The PSI was primarily governed by a rise in the HR and less by the internal temperature in the tested groups (Table 4). No significant differences were found for the *f*HR PSI and *f*Tty PSI between T1 and T2 (Table 4).

### 3.4. The Effect of Heat Acclimation on Changes in Hematological Biomarkers Following Exercise

**Figure 2:**
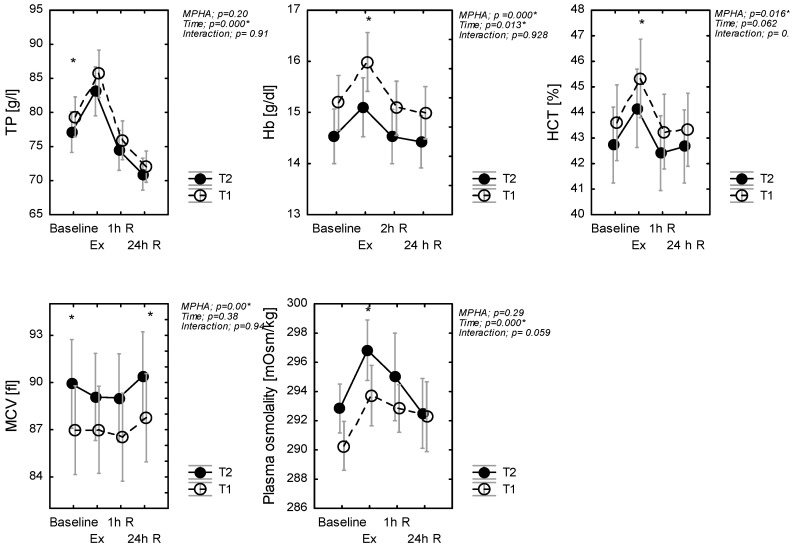
Plasma protein (TP), hematocrit (HCT), and hemoglobin (Hb) concentration and plasma osmolality, mean red cellular volume (MCV); at rest (baseline), in response to exercise (Ex) (60-min exercise at constant work rate (60% power max) and during recovery (1 h R; 24 h R), before (T1) and after MPHA (T2). Data are presented as mean ± SD, *n* = 10 for all data; * significance of differences between control (T1) and heat acclimated person (T2) in the same time of testing.

A main effect of treatments was evidenced for hematocrit HCT (*p* = 0.016, η^2^*p* = 0.12), MCV (*p* = 0.000, η^2^*p* = 0.48), and hemoglobin (*p* = 0.04, η^2^*p* = 0.28) concentration just as a main effect of exercise, but no evidence of an interaction effect was found (Figure 2). Pairwise comparisons showed that HCT and Hb increased (whereas MCV decreased) during exercise in T2, in comparison to T1 conditions (Figure 2). There was no significant effect of MPHA on TP (*p* = 0.20), but there was a significant effect of time (*p* < 0.001, η^2^*p* = 0.46), but again no interaction effect (*p* = 0.91). Post hoc analysis revealed that TP in T2 was lower at rest during and after the exercise test. With recovery, %ΔTP (1h) increased and then decreased in a similar manner in T1 and T2 (Figure 2).

Baseline OsM was similar between T2 and T1 (*p* = 0.243). Across exercise, OsM increased by 4 and 5 mOsm·kg^−1^ in T1 and T2, respectively. As such, the increase in post-exercise %ΔOsM was similar in T1 and in T2 (*p* = 0.64). There was a significant effect of time (*p* < 0.001, η^2^*p* = 0.16) for OsM. Plasma osmolality immediately increased after exercise but decreased during recovery.

### 3.5. Changes in Plasma Volume Following Exercise

**Figure 3:**
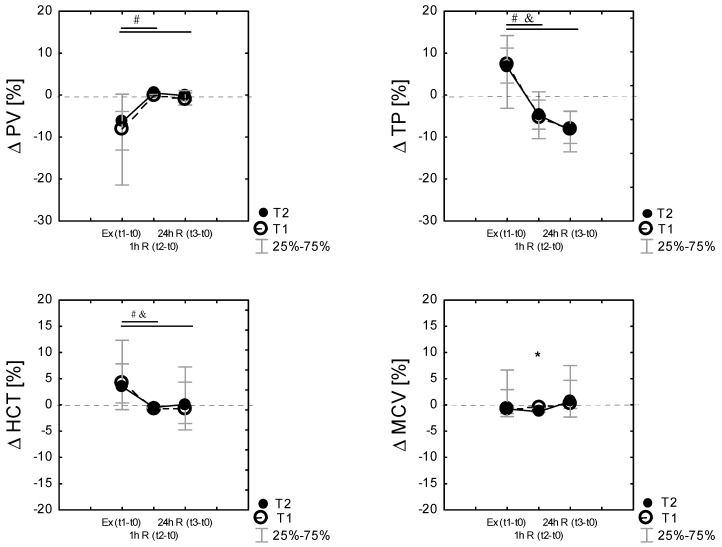
Differences (% Δ) related to baseline (t0) of plasma volume (% ΔPV), total proteins (%Δ TP), hematocrit (%Δ HCT), mean red cell volume (% Δ MCV), immediately after exercise test Ex (t1−t0), as well as 60 min 1 h R (t2−t0)) and 24 h after the exercise 24 h R (t3−t0)) in the control (T1) and heat acclimated (T2) cross-country skiers. Results are presented as median and IQR; 25%–75%, *n* = 14 for all data. * Significance of differences between control (T1) and heat-acclimated subjects (T2) in the same time of testing, *p* < 0.05. (by the Wilcoxon test). Note: Friedman’s ANOVA results: (% ΔPV): T1 group: χ^2^ df = 2, *n* = 14 = 9,78, *p* = 0.007; T2 group: χ^2^ df = 2, *n* = 14 = 14.0, *p* = 0.000; Friedman’s ANOVA results (%Δ TP): T1 group: χ^2^ df = 2, *n* = 14 = 32.7, *p* = 0.000; T2 group: χ^2^ df = 2, *n* = 14 = 25.0, *p* = 0.000; Friedman’s ANOVA results: (% ΔHCT): T1 group: χ^2^ df = 2, *n* = 14 = 20.03, *p* = 0.000; T2 group: χ^2^ df = 2, *n* = 14 = 13.6, P = 0.001; Friedman’s ANOVA results: (% ΔMCV): T1 group: χ^2^ df = 2, *n* = 14 = 10.3, *p* = 0.001; T2 group: χ^2^ df = 2, *n* = 14 = 8.19, P = 0.016, # *p* < 0.05 vs. the respective exercise value, by the Wilcoxon test in T1, and *p* < 0.05 vs. the respective exercise value, by the Wilcoxon test in T2.

Plasma volume shifts were evident following exercise. PV shifts from baseline were calculated following each exercise session. Exercise and recovery significantly affected %ΔPV during the experiment in T1 (χ^2^ 9.78; *p* = 0.007 in Friedman’s ANOVA nonparametric test) and in T2 (χ^2^ = 14. *p* = 0.000 in Friedman’s ANOVA nonparametric test) conditions. Compared to the baseline (t0), PV was reduced post-exercise (Ex (t1–t0)) in T1 by −7.92% [−21.6; −3.9] and in T2 by −6.36% [−13.09; 0.23]. The reduction in the plasma volume after exercise was not different between the conditions (*p* > 0.05). Finally, there were no differences in the percentage change in the plasma volume after 24 h recovery between T1 and T2 (Figure 3).

Percentage changes in MCV, HCT, TP, and PV following exercise were analyzed by time points. Figure 3 displays the mean percent changes (median; the 25th and 75th percentiles) from the pre-exercise baseline values, where it can be identified that ΔMCV, ΔHCT, ΔTP, and ΔPV change in similar patterns in both T1 and T2. Friedman’s ANOVA nonparametric test showed a significant effect of time (exercise, recovery) in both T1 (*p* < 0.001) and T2 (*p* < 0.001) for all tested variables. Percentage changes in TP following exercise correlated with changes in percentage changes in HCT and PV (Figure 3).

To obtain more information about the main functions of plasma proteins, namely the maintenance of the colloid osmotic capacity, we related the percentage changes in TP (%TP) to the percentage changes in PV (%PV) by adding up %ΔPV and %ΔTP. As expected, value (E), i.e., that no protein enters or leaves the intravascular space (IVS), was marked as E = 0 [63]. The E values characterized the shift of proteins into the intravascular space in response to MPHA and exercise but not during recovery.

There was a significant correlation between the percentage change in PV and the percentage change in average TP in T2, and there was a significant negative correlation between the percentage change in PV and the percentage change in average HCT in T1 and T2 (Table 5) during the exercise test. Apart from that, there was a negative and significant correlation between the percentage change in PV and the percentage change in average MCV following exercise in T1 and T2 and a significant positive correlation between the percentage change in PV and the percentage change in average MCV during recovery (Table 6). The PSI was correlated with the percentage change in %ΔTP (Table 6) only in T2 conditions.

## 4. Discussion

In this study, thermal, physiological, and hematological indices were assessed in elite athletes at rest and in response to an hour-long submaximal exercise test performed under thermoneutral conditions before and after a series of ten sauna baths. The main findings of this study are as follows:

(1) The MPHA model used in our experiment was insufficient to produce a typical phenotype indicative of heat adaptation. The results of the study showed that the athletes’ use of a series of sauna baths during the transition period of a training cycle caused only partial acclimation to heat; (2) characteristics of acclimation to heat, after completing a series of sauna baths, were manifested by lower heart rate at rest (by ~8 bs/min), lower SDP at rest, increased PV (by 7.42%), decreased TP in plasma (by 5.8%), and increased MCV (by 4.11%), but without a significant decrease in the core temperature or body and skin temperature; (3) the adaptive changes achieved after MPHA mainly affected the functions of the circulatory system and resulted in a general reduction in physiological strain (PSI) during physical effort performed under thermoneutral conditions. Lower PSI was correlated with a lower reduction in the plasma volume and lower plasma protein deficiency in response to exercise; (4) acclimation (MPHA) did not differentiate the time course of changes in the plasma volume after exercise but showed an effect on the amount of water shifts from cells to the extracellular space and on the strength of the relationship between changes in %ΔPV relative to changes in plasma %ΔTP and the mean % ΔMCV during the recovery period.

### 4.1. Thermal and Physiological Changes after a Series of Ten Sauna Baths

A lower resting and exercising body temperature is a common and rapidly adapting phenotypic attribute of heat acclimation [12,68]. However, less information is available on whether this phenotype can be found in elite athletes [10]. With the exception of lower HR at rest, a lower HR response during the last minutes of exercise, and an increase in the plasma volume and MCV, only subtle adaptive changes involving body temperature characteristics were observed in this study (Table 3). The presented model of heat adaptation in elite athletes did not cause significant changes in the core and skin temperatures at rest, although the subjects’ Tty temperature remained lower by 0.18 °C after a series of ten sauna baths. This result is in line with earlier studies that the passive model of heat acclimation PMHA (passive exposure to 33 °C for 7 consecutive days elicited a decrease in the core temperature by −0.13−0.14 °C [40]. A reduction of 0.3 °C in Tre was reported in previous studies using controlled hyperthermia [13,69] or using passive acclimation protocols (HWI) after exercise [46]. Often [4,29,68,70] but not always, changes in internal temperature were reported [13,71] after thermal acclimation. Considering the decrease mainly in HR and the weak and insignificant effect of MPHA on body temperature after the acclimation period in our study, it is possible that the athletes experienced a less optimal adaptation, mainly cardiovascular adaptation. The most common physiological adaptation recorded after HA is a reduction in the heart rate (HR) [20]. Physiological systems usually adapt to the specifics of an adaptive stimulus, and in the overall scheme, in principle, different models (passive, active) can induce qualitatively similar but quantitatively different adaptive changes.

Moreover, to develop the typical heat adapted phenotype, constant thermal forcing should be applied, as the forcing function decreases with the progress of adaptation. The passive medium-term heat acclimation model used in this study could be too weak an adaptive stimulus for elite athletes who had already developed some features of adaptation to heat in the training process, and this entails lower effectiveness of forcing systemic changes. The low effectiveness of MPHA in developing changes that involve temperature characteristics could also be due to the fact that in the current research, the athletes did not keep to the time regime regarding the core temperature >38.5 °C during sauna bathing (the baths were interrupted by periods of body cooling). It is known that heat acclimation typically consists of repeated daily heat stress exposures, with the exposure duration commonly being 60–90 min [4,55] and involving daily or alternate days of heat stress over a period of 5–16 days, whereby Tc, Tsk, and sweat rate are elevated for 1–2 h [14]. Therefore, the sauna bathing applied in our study may have been an insufficient period to induce the necessary thermoregulatory adaptations required to decrease the core temperature. Recent research by Corbett et al. [11] reported no association between thermal stress (time spent > 38.5 °C) during CHI sessions and the development of changes in Tc.

Another obstacle to forcing effective development of attributes of the heat-adapted phenotype could be the respondents’ high level of training. The athletes were characterized by very high functional performance and the maximum aerobic capacity (VO_2_max; 64 mL/kg/min). There is evidence that individuals with high aerobic capacity (VO_2_max) can be partially acclimated [18,72], possibly due to the occurrence of some training adaptations (e.g., hypervolemia). Taylor [28] suggests that highly trained athletes have less adaptive potential compared with untrained or moderately trained participants, and the higher the background of adaptation, the lower the adaptation response. On the other hand, Garrett et al. [10] showed that a daily 90-min isothermic HA protocol was an adequate stimulus for heat adaptation and improved cardiovascular stability in highly trained athletes. Moss et al. [73] suggest that a 5-day 60-min isothermic HA regimen provides a sufficient thermal stimulus to elicit beneficial adaptations to reduce physiological and perceptual strain while exercising in heat in ultra-endurance runners. Furthermore, people with a high VO_2_max can acclimate faster than people with lower VO_2_max [74]. Taking into account the apparent independence between some indicators characterizing HA, Corbett et al. [11] showed that baseline VO_2_max (absolute or relative) was not related to the initial thermophysiological responses to exercising in heat nor to the magnitude of the adaptive responses following the HA intervention. The discrepancy in the researchers’ findings also concerns the differences in individual and periodically changed (training people’s) sensitivity to interactions used to develop the characteristics of thermal adaptation [37,75], when the increased heat load of the body can [76] or may not result in triggering [77] short-term adaptive changes in the cardiovascular and hematological systems [11,28].

This research was conducted during a transitional phase of the annual training period. This period is dedicated to athletes’ regeneration and is characterized by the lowest volume and intensity of exercises in the training macrocycle, which promotes regeneration of athletes’ bodies. We hypothesized that sauna-based heat acclimation could reduce the amount of physiological stress in response to exercise. The PSI index and the share of thermoregulatory and cardiovascular stresses in the general physiological stress during submaximal exercise before and after MPHA were assessed. The adaptive changes achieved after MPHA were manifested during exercise by a general reduction in physiological strain (PSI). The assessed PSI index reflects the total load on the thermoregulatory and circulatory systems [34]. The principle behind PSI is an evaluation of the physiological strain resulting from the cardiovascular and the thermoregulatory systems. Under thermoneutral conditions, the load on the thermoregulation system depends on the metabolic production of heat H_prod_ [78]. Changes in the core temperature and sweating during exercise in a neutral climate are determined by H(_prod_), mass, BSA (not VO₂ peak), and the efficiency of heat dissipation, and changes in the cardiovascular system depend on the relative workload, type of effort, volume, and availability of blood for the cardiovascular function. The PSI has demonstrated validity in discriminating between levels of heat strain during laboratory experimental manipulations of environmental heat, heat acclimation status, aerobic fitness status, hydration status, and exercise intensity levels [34,79]. During both exercise interventions (before and after a series of sauna baths), mean absolute workload % power max, W/kg (absolute H_prod_) [72] were similar, and still, there was a tendency to lower physiological strain during exercise in subjects after a series of sauna baths (*p* = 0.052, ES = 0.87). Generally, heat acclimation results in numerous adaptations that reduce physiological strain, thus leading to enhanced submaximal and maximal aerobic exercise performance in the heat [4]. According to the categorization of PSI [34], both groups (T1 and T2) have experienced a moderate level of physiological strain by the end of one hour of the exercise test. Moreover, it was found that in the test conditions, the share of the circulatory component (*f*HR) in determining the physiological strain (PSI) was greater than that of the thermoregulation system (*f*Tty) and did not significantly differ before and after MPHA. The fractional contribution of HR to PhSI (*f*HR) was quite high because the relative rate of rise in heart rate exceeded the initial rate of rise in the core temperature [80]. A greater share of the circulatory strain than of the thermoregulatory one during exercise was also noted in previous studies of athletes who exercised until fatigue under conditions of moderate ambient temperature. The share of the circulatory component *f*HR in the contribution of the cardiovascular fraction to the PSI (circulatory strain) accounted for approx. 70–80% of the PSI volume [61] in highly trained athletes (runners and cyclists). In the current study in all subjects, the circulatory strain (*f*HR) was the major component (0.76–0.77) determining the overall physiological strain during the submaximal exercise test performed before and after MPHA by elite athletes (cross-country skiers) in a thermoneutral environment. It is noteworthy that after a series of sauna baths, the heart rate during exercise was ~5 bpm lower than in the control, with a reduction of ~8 bpm recorded at rest. The reduction in HR after MPHA is consistent with the results of studies in which lower HR responses were reported after the HA program [5,12], and those in which hematological and circulatory manifestations of heat adaptation were associated with increased plasma volume [8,20] in men of various training statuses (healthy, active, well trained, and competitive) following heat chamber, sauna, and HWI protocols with 40–120 min of heat exposure [39,41,43,46].

### 4.2. Passive Mild Heat Acclimation and the Human Plasma Volume Changes

The present study has demonstrated that sauna bathing following normal training induced moderate PV expansion in well-trained cross-country skiers after ten exposures to sauna. Physiological strain was correlated with %ΔPV and %ΔTP but not with HRR, which confirms the previously noted strong association between cumulative strain and the magnitude of PV in Akerman’s study [9]. Plasma volume increase after acclimation (ΔPV) increases the ventricular filling pressure and the stroke volume, which helps maintain the cardiac output and reduces HR when exercising in heat [25]. The amount of plasma volume expansion after acclimation to heat is different and depends on the technique and time of measurement, population, the number of heat exposures, protein and carbohydrate supplementation, and the level of the thermal stimulus [20,27,70,81] as well as background endurance training and/or baseline PV.

The results of the current research indicate that MPHA caused a relatively small increase in PV (7.42%) at rest, while others generally reported a significant increase (5–16%) in PV after HA: [26]—about 5%, [41]—7%, [43]—18%), even in trained individuals (VO_2_max: ~60 mL/min/kg). Senay et al. [25] showed an increase in the plasma volume after a 10-day HA program, ranging from ~8 to 33%, which was consistent with observations by Racinais et al. [71], who also noted an increase in the plasma volume after thermal acclimation, indicating high inter-subject variability in the adaptive response to the 6-day heat acclimation program (including the change (∆) in the plasma volume from −10 to +20%). Results of recent studies using the standard 10-day laboratory HA intervention also showed an increase in PV and a broad spectrum of post-HA adaptive responses including an increase in the plasma volume [77,82]. Studies using regular passive acclimation treatments indicate that exposure to the sauna after exercise is an effective and efficient way to initiate PV expansion compared to traditional heat acclimation, which includes training in hot ambient conditions [2,5,13,29], although its effectiveness is lower than that of training, especially training in the heat.

A classic explanation of the reasons for an increase in PV after HA (acclimation hemodilution) is proposed by Senay [83], who believes this is due to an increased tendency for protein to remain in the intravascular space. The effect of the increased content of intravascular protein is an increase in oncotic pressure and, therefore, the possibility of greater net movement of fluid from the interstitial space to the intravascular space and a greater filtration of the vascular fluid from increased capillary hydrostatic pressure associated with an increased cutaneous blood flow in response to high skin temperatures (>38.8 °C) [26,47]. However, the results of our research indicated that the concentration of TP was lower after MPHA, and the blood plasma proteome profile did not significantly differ before and after acclimation to heat [54]. It appears that most of the increase in plasma protein content with classical heat acclimation is due to the metabolic (exercise) stimulus, rather than to environmental and body heating [83,84]. Recently, using differential scanning calorimetry (DSC) in analyzing plasma heat capacity changes, Mourtakos et al. [85] indicated that 5 days of exhaustive physical exercise of highly trained individuals enhanced the thermal stability of plasma albumin shifting its denaturational transition to a higher temperature. In our previous study, using DSC to assess potential alterations in the plasma heat capacity and to register characteristic post-exercise changes in the profile of thermal denaturation transition of serum after the sauna treatments [86], we indicated that the alterations in the plasma proteome denaturational profiles were not persistent. In accordance with Rocker et al. [65], the change in intravascular plasma protein mass (TPP) was estimated. Only during exercise and in the initial period of regeneration the influx of proteins into IVS was increased, and it gradually decreased during the recovery period (Figure 3). Net total protein gained during and after the exercise test in group T2 was found not to be significantly different from that gained in control (T1). We reported that similar quantities of protein were added to the vascular volume in both the control and the MPHA groups after the exercise test; however, changes in the athlete’s blood serum proteome, conditioning the modification of serum DSC profiles in the session held after sauna treatments, were stronger than those in the session not preceded by treatments [86].

The results of our research show a significant relationship between changes in %ΔPV and changes in %ΔTP in response to physical effort, which is consistent with the post-exercise increase in plasma colloid osmotic strength and the retention of water in the vascular space. Intravascular protein and fluid shift during exercise may alter the subsequent plasma volume change during the recovery period [87]. In the current research, there was a significant negative correlation between %ΔPV and %ΔMCV in response to exercise and a positive one at 24 h recovery in both groups (Table 6). Acclimation (MPHA) significantly differentiated the behavior of changes in the plasma volume after exercise and had an impact on the amount of water shifts from cells to the extracellular space and on the strength of the relationship between changes in the plasma volume (%ΔPV) relative to changes in plasma protein concentration (%ΔTP) and the mean red cellular volume (%ΔMCV) during the restitution period. The rapid recovery of the blood volume after exercise was initiated by a rapid increase in total circulating plasma proteins as shown in the present study and other studies [35,88]. This process of expanding plasma was aided by water shifts from the cells and a reduction in the mean MCV volume. According to Périard et al. [20], increasing PV and MCV is an important factor contributing to the improvement in endurance performance, ensuring greater stability of the plasma volume, greater filling of the ventricles, and consequently, the preservation of greater stroke volume. The current findings suggest that the changes achieved after MPHA associated with moderate increases in PV and MCV may help to maintain the plasma volume stability during submaximal exercise and the cardiovascular stability, thus promoting a smaller overall physiological strain during exercise under temperate conditions. The relationship between the changes in %ΔPV and a change in %ΔMCV and %ΔTP as well as between PSI and %ΔTP in response to exercise confirms that lower physiological strain on the body during exercise seems to be achieved under conditions of better PV preservation in the vascular bed [26]. The current findings suggest that the changes achieved after MPHA may help to maintain the plasma volume stability during submaximal exercise and the cardiovascular stability, thus promoting a smaller overall physiological strain during exercise under temperate conditions.

### 4.3. Limitation

The current study has some limitations. Firstly, the examined group of athletes were, namely, elite, highly trained cross-country skiers in the recovery phase. As Coyle et al. [89] maintain, untrained individuals may benefit more from moderate PV expansion, while inducing hemodilution in trained individuals with high PV expansion not accompanied by a significant increase in red blood cell count may not be of much benefit to the athletes. Although we assumed some improvement in system functions after the passive model of thermal adaptation in athletes using the sauna, less trained participants may show better symptoms of adaptation to heat after a series of sauna baths. Secondly, the effectiveness of inducing functional changes after adaptation may be different in the competitors’ preparation and competitive phases and depend on the maintenance of euhydration and the adequate fluid-replacement strategies recommended for rehydration in each of the training period. Unfortunately, neither macronutrient intake, total energy intake/daily energy expenditure, nor the electrolyte consumption and/or balance were monitored in this study.

## 5. Conclusions

Summing up, it can be concluded that the results of the conducted research did not provide sufficient evidence to confirm the occurrence of (full) thermal adaptation in a group of elite cross-country skiers after MPHA in the transition phase of the training program. Improvements in PSI and Tb during exercise were noted after MPHA; however, these did not reach the predefined threshold of significance. Adaptive changes developed after MPHA resulted in an increase in PV and an improvement in the cardiovascular function and the plasma volume stability during and immediately after completion of submaximal exercise, which may indicate that the use of a regular sauna bath strategy during the transition period of the training macrocycle may support the body’s recovery process after exercise in elite cross-country skiers. Regarding the recovery process after exercises, a large individual variability was observed in both sessions, and these athletes needed more time to reverse the changes in serum caused by intense exercises performed after a series of 10 sauna treatments.

## Figures and Tables

**Table 1:** Anthropometric characteristics of the recruited participants.

Indicators	$\bar{X}$ ± SD	Min	Max
Age [years]	21.2 ± 2.99	18	27
Body height [cm]	178.8 ± 4.12	167	183
Body mass [kg]	70.6 ± 6.0	59	80.6
BSA [m^2^]	1.86 ± 0.13	1.71	2.18
BMI [kg m^−2^]	22.17 ± 1.83	18.4	24.9
FM [kg]	5.94 ± 2.56	1.3	11
FFM [kg]	50.47 ± 13.17	36.2	71.5
TBW [kg]	49.6 ± 6.1	43.0	60.7
Training status [years]	9.5 ± 10.5	7	12

Legend: BSA—body surface area; BMI—body mass index; FM—total body fat; FFM—total fat free mass; TBW—total body water; the data are represented as mean ± SD; *n* = 16 for all data.

**Table 2:** Key performance characteristics of the research participants from preliminary exercise testing (*n* = 16).

Indicators	$\bar{X}$ ± SD	Min	Max
VO_2max_ [mL·kg^−1^·min^−1^]	64.5 ± 6.33	53	73
HR_max_ [bs·min^−1^]	191 ± 8.72	171	206
Power_max_ [W]	395.7 ± 9.1	310	497
Power_max_ [W·kg^−1^]	5.68 ± 0.5	5.3	6.2
RER_max_	1.05 ± 0.6	0.95	1.21
MET_max_	17.9 ± 1.9	14.9	21.8
Ve _max_ [L·min^−1^]	156 ± 25.1	120.4	198
LA _max_ [mmol·L^−1^]	9.83 ± 1.84	6.7	13.5
HR_AT_ [bs·min^−1^]	173.3 ± 7.7	160	183
V_AT_ [km·h^−1^]	13.6 ± 0.9	12	14
G% _AT_ [%]	1.25 ± 1.6	1	5

Data are presented as mean ±SD, min, max; *n* = 16 for all data. Legend: VO_2max_—maximal oxygen uptake; HR_max_—maximal heart rate; Power_max_—maximal power rate; RER_max_—maximal respiratory exchange ratio; MET_max_—maximal metabolic equivalent of energy; Ve_max_—maximal ventilation; LA_max_—maximal blood lactate concentration; HR_AT_—heart rate at the individual anaerobic threshold; V_AT_—treadmill velocity at the individual anaerobic threshold; G%_AT_—treadmill inclination at the individual anaerobic threshold. All athletes were subjected to the procedures of passive whole-body hyperthermia during the transition phase of their training. The total time individuals spent in sauna sessions was in the range 32–52 min.

**Table 3:** Average exercise workload and average of physiological indicators at rest and at the end of the EET test before (T1) and after MPHA procedures (T2) with corresponding statistical significance (ANOVA output) and comparisons of interest.

Variables	T1 (*n* = 14) X ± SD	T2 (*n* = 14) X ± SD	*p*	ES	Effect of MPHA; p; η^2^p Effect of Exercise; p; η^2^p Interaction; p; η^2^p
VO_2 rest_ [mL·kg^−1^·min^−1^]	0.74 ± 0.04	0.75 ± 0.05	0.28	0.22	MPHA; *p* = 0.08 Exercise; *p* = 0.001; 0.95 Interaction; *p* = 0.57
VO_2 last_ [mL·kg^−1^·min^−1^]	3.67 ± 0.1	3.68 ± 0.12	0.41	0.09	
HR _rest_ [bs·min^−1^]	66.5 ± 11.2	58.5 ± 3.78 ***	0.001	1.08	MPHA; *p* = 0.006; 0.44 Exercise; *p* = 0.001; 0.95 Interaction; *p* = 0.57
HR _last_ [bs·min^−1^]	167.9 ± 2.6	162.3 ± 3.4 ***	0.001	1.23	
T_ty rest_ [°C]	36.5 ± 0.6	36.3 ± 0.4	0.31	0.40	MPHA; *p* = 0.11 Exercise; *p* = 0.000; 0.58 Interaction; *p* = 0.57
T_ty last_ [°C]	37.6 ± 0.6	37.3 ± 0.4	0.11	0.63	
Ts_Ch rest_ [°C]	33.8 ± 1.4	33.2 ± 1.3	0.09	0.44	MPHA; *p* = 0.17 Exercise; *p* = 0.84 Interaction; *p* = 0.72
Ts_Ch last_ [°C]	33.8 ± 1.4	33.2 ± 2.1	0.3	0.34	
Ts_F rest_ [°C]	32.9 ± 1.0	32.2 ± 1.4	0.09	0.62	MPHA; *p* = 0.79 Exercise; *p* = 0.001; 0.53 Interaction; *p* = 0.68
Ts_F last_ [°C]	33.3 ± 1.5	33.8 ± 1.3	0.08	0.35	
Ts_Th rest_ [°C]	31.2 ± 1.3	31.3 ± 0.8	0.38	0.15	MPHA; *p* = 0.59 Exercise; *p* = 0.08 Interaction; *p* = 0.21
Ts_Th last_ [°C]	32.8 ± 1.4	32.4 ± 1.6	0.40	0.26	
$\bar{T}$_b rest_ [°C]	35.6 ± 0.7	35.5 ± 0.4	0.53	0.31	MPHA; *p* = 0.054; Exercise; *p* = 0.000; 0.65 Interaction; *p* = 0.84
$\bar{T}$_b last_ [°C]	36.7 ± 0.5	36.4 ± 0.7	0.17	0.63	
$\bar{T}$_SK rest_ [°C]	32.8 ± 1.2	32.4 ± 0.8	0.25	0.36	MPHA; *p* = 0.45 Exercise; *p* = 0.001; 0.3 Interaction; *p* = 0.83
$\bar{T}$_SK last_ [°C]	33.4 ± 1.3	33.2 ± 1.4	0.68	0.66	
SBP _rest_ [mmHg]	138.3 ± 19.3	125.5 ± 6.2 *	0.04	0.96	MPHA; *p* = 0.03; 0.25 Exercise; *p* = 0.45 Interaction; *p* = 0.92
SBP _last_ [mmHg]	136.8 ± 11.6	140.4 ± 14.2	0.65	0.28	
DBP _rest_ [mmHg]	71.7 ± 2.9	70.8 ± 10.7	0.73	0.14	MPHA; *p* = 0.34; Exercise; *p* = 0.51 Interaction; *p* = 0.74
DBP _last_ [mmHg]	70.7 ± 5.3	71.5 ± 8.3	0.16	0.11	
Power [W] _last_	237.0 ± 36.5	237.5 ± 35.3	0.94	0.01	
Power [W·kg^−1^] _last_	3.39 ± 0.04	3.41 ± 0.07	0.19	0.36	

Data are means ± SD. HR—heart rate; Tty—internal (tympanic) temperature; T_SK_—mean skin temperature; Ts_Ch_—chest skin temperature; Ts_F_—forearm skin temperature; Ts_Th_—thigh skin temperature; Tb—mean body temperature; SDP—systolic blood pressure; DBP—diastolic blood pressure; VO_2_—oxygen uptake; rest—resting values; last—end values; *n* = 14 for all data except for skin and mean body temperature (*n* = 10) in T1; (*n* = 14) for all data in T2. * *p* < 0.05; ** *p* < 0.005; *** *p* < 0.001 represents a significant difference within the group between session 1 (control; T1) and 2 (after MPHA acclimation; T2). Two-way analysis of variance (ANOVA) and Tukey’s post hoc test were used to identify differences between T1 and T2.

**Table 4:** Differences (Δ) in physiological indicators in response to the exercise EET test with the corresponding physiological strain and cardiovascular and thermal fraction of physiological strain for (T1) and (T2).

Variables	T1 (*n* = 14) X ± SD	T2 (*n* = 14) X ± SD	*p*	ES
Δ T_b_ [°C]	0.89 ± 0.51	0.62 ± 0.64	0.243	
Δ T_SK_ [°C]	0.57 ± 1.04	0.78 ± 1.03	0.7	
Δ BM [kg]	−1.06 ± 0.6	−1.13 ± 0.81	0.19	
Δ T_ty_ [°C]	1.12 ± 0.8	1.0 ± 0.6	0.06	
HRR [bs·min^−1^]	99.4 ± 6.19	104.1 ± 8.56	0.35	
*f*T (PSI)	0.24 ± 0.11	0.22 ± 0.14	0.22	
*f* HR (PSI)	0.76 ± 0.12	0.77 ± 0.11	0.78	
PSI	6.03 ± 1.11	5.27 ± 0.62	0.052	0.87

Legend: ΔTb—mean body temperature change in response to exercise; ΔTsk—mean skin temperature change in response to exercise; ΔBM—body mass change [kg]; ΔTty—tympanic temperature change in response to exercise; *f*HR—the contribution of the cardiovascular fraction to the PSI; *f*T_ty_—the contribution of the thermal fraction to the PSI; PSI—physiological strain index; HRR—heart rate reserve.

**Table 5:** Spearman correlation coefficient (“r”) between the percentage change in plasma volume, plasma protein changes, and hematological and osmolality changes in response to exercise test (Δ%) and during recovery before (T1) and after MPHA (T2) in elite cross-country skiers.

	T1			T2		
Variables	Δ PV%	Δ TP%	Δ HCT [%]	Δ PV%	Δ TP%	Δ HCT [%]
Δ PV [%]		−0.44			−0.71 *	
Δ TP [%]	−0.44			0.71 *		
Δ HCT [%]	−0.97 *	0.23		−0.98 *	0.73 *	
Δ OSM [%]	0.11	0.39		0.03	−0.05	
Δ MCV [%]	−0.83 *	0.2	0.96 *	−0.89 *	0.69 *	0.93 *
Δ MCV [%] 1 h R (t2–t0)	0.35	0.13		0.03	−0.04	
Δ MCV [%] 24 h R (t3–t0)	0.85 *	-0.25	0.88 *	0.52 *	0.07	0.88 *

* a significant Spearman correlation coefficient *p* < 0.05.

**Table 6:** Spearman correlation coefficient (“r”) between physiological strain (PSI) and the percentage change in plasma volume (PV), osmolality (OSM), protein (TP), and hematocrit (HCT) and mean red cell volume (MCV) changes (Δ%) in response to exercise before (T1) and after MPHA (T2) in elite cross-country skiers.

	T1	T2
Variables	PSI	PSI
Δ PV [%]	−0.37	−0.49
Δ TP [%]	−0.01	0.76 *
Δ HCT [%]	0.33	0.41
Δ OSM [%]	0.09	0.19
Δ MCV [%]	0.08	0.28

* a significant Spearman correlation coefficient (*p* < 0.05) represents a significant difference between session 1 (control; T1) and 2 (after MPHA acclimation; T2).

## Data Availability

The data presented in this study are available on request from the corresponding author.
